# Association of tumor mutational burden and tumor-infiltrating lymphocytes with second primary cancer after complete resection of non-small cell lung cancer

**DOI:** 10.3389/fonc.2026.1773288

**Published:** 2026-09-17

**Authors:** Marie-Pier Gauthier, Maryam Karimi, Stefan Michiels, Lesley Seymour, Elisabeth Brambilla, Thierry Le-Chevalier, Jean-Charles Soria, Robert Kratzke, Stephen L. Graziano, Ramaswamy Govindan, Ming-Sound Tsao, Frances A. Shepherd

**Affiliations:** 1 Princess Margaret Cancer Centre, University Health Network, Toronto, ON, Canada; 2 Bureau de Biostatistique et d’Epidémiologie, Gustave Roussy, Université Paris-Saclay, Villejuif, France; 3 Oncostat U1018, Inserm, Université Paris-Saclay, Equipe Labellisée Ligue Contre le Cancer, Villejuif, France; 4 Canadian Cancer Trials Group and Queen’s University, Kingston, ON, Canada; 5 Department of Pathology, Institut Albert Bonniot, Hopital Albert Michallon, Grenoble, France; 6 Department of Medical Oncology, Gustave Roussy, University Paris-Saclay, Villejuif, France; 7 Division of Hematology-Oncology-Transplantation, University of Minnesota, Minneapolis, MN, United States; 8 Medical Oncology, SUNY Upstate Medical University, Syracuse, NY, United States; 9 Division of Medical Oncology, Department of Medicine, Washington University School of Medicine, St. Louis, MO, United States; 10 Department of Laboratory Medicine and Pathobiology, University of Toronto, Toronto, ON, Canada; 11 Department of Medicine, Division of Medical Oncology, University Health Network, Toronto, ON, Canada

**Keywords:** biomarkers, Lung Adjuvant Cisplatin Evaluation (LACE), non-small cell lung cancer (NSCLC), second primary cancer (SPC), tumor mutational burden (TMB), tumor-infiltrating lymphocytes (TILs)

## Abstract

**Introduction:**

The risk of developing second primary cancers (SPC) after complete surgical resection of non-small cell lung cancer (NSCLC) has been estimated at 1-2% per patient-year. We investigated whether tumor genomic instability, assessed by tumor mutational burden (TMB), and host immune response, assessed by tumor-infiltrating lymphocytes (TILs), were associated with the development of SPC.

**Methods:**

Data from three randomized trials included in the Lung Adjuvant Cisplatin Evaluation-Biomarker (LACE-Bio) meta-analysis were used. TMB and TILs were assessed on FFPE specimens. TMB was categorized into tertiles (high >7.8 mutations/MB, moderate >4 to ≤7.8 mutations/MB, and low ≤4 mutations/MB), and TILs were classified as marked vs. other. Associations between biomarkers and competing endpoints (SPC, and death without developing SPC) were evaluated using Fine and Gray sub-distribution hazard models, stratified by trial and adjusted for treatment, age, sex, tumor stage, nodal stage, histology, performance status, and surgery type.

**Results:**

TMB and TILs assessments were available for 879 patients with complete clinical covariates. Marked TILs was observed in 85 patients. During follow-up, 47 SPCs and 392 deaths without SPC were observed. No clear association was found between TMB or TILs and the cumulative incidence of SPC. In contrast, low TMB was associated with a higher cumulative incidence of death without SPC compared with moderate TMB (multivariable subdistribution hazard ratio (sHR) = 1.35 [95% confidence interval (CI), 1.06-1.72], p = 0.02). Marked TILs was associated with a lower cumulative incidence of death without SPC, although this association did not reach statistical significance after adjustment (multivariable sHR = 0.71 [95% CI, 0.46-1.10], p = 0.12).

**Conclusion:**

This study is the first to evaluate associations between tumor genomic instability and host immune response, and competing endpoints of SPC and death without SPC in patients with completely resected NSCLC. No strong association was observed with SPC, whereas low TMB (compared with moderate TMB) was associated with the competing endpoint of death without developing SPC. Given the limited number of SPC events, these findings require confirmation in larger, independent cohorts of patients with early-stage lung cancer.

## 1 Introduction

Lung cancer remains the leading cause of cancer-related death worldwide (1). Non-small cell lung cancer (NSCLC), which accounts for about 80-85% of all lung cancers, now reaches 5-year survival rates around 60-70% in patients with resected stage I-II disease (2), whereas 5-year survival for advanced or metastatic stages remains low, around 10-15%, although it can reach 20-30% in selected patients treated with modern immunotherapy or targeted therapies (3). As survival improves for patients with early-stage disease, long-term survivorship issues are becoming increasingly important. In particular, patients treated for lung cancer may develop additional malignancies during follow-up, including a new, unrelated cancer, referred to as a second primary cancer (SPC).

The risk of developing an SPC after complete surgical resection of NSCLC has been reported to be approximately 1-2% per patient-year (4). Among patients surviving free of cancer for two or more years, the relative risk for any SPC compared with that in the general population is 4.4 [95% confidence interval (CI), 2.5-7.2], with a relative risk of 16 (95% CI, 8.4-27) for a second primary non-small-cell lung cancer (5). This elevated risk remains over time, particularly among smokers.

Studies have shown the continued tobacco use after cancer diagnosis as a well-established risk factor for the development of SPCs in several tobacco-related malignancies, including lung, head and neck, and bladder cancers (6). Despite this increased risk, due to limited data, few molecular biomarkers have been investigated to identify patients with resected NSCLC who may be at higher risk of developing SPC. Within the Lung Adjuvant Cisplatin Evaluation-Biomarker (LACE-Bio) group, SPCs were reported to occur more frequently in patients with *KRAS*-mutated lung cancers (7); however, similar associations were not observed for other biomarkers evaluated by this group (8–16).

Lung cancer is characterized by a high tumor mutational burden (TMB), defined as the total number of somatic mutations present in a tumor genome, largely reflecting the mutagenic effects of tobacco exposure, although this association varies across histological subtypes and is generally stronger in smoking-related tumors such as squamous cell carcinoma (17). In previous analyses from the LACE-Bio group, high nonsynonymous TMB was associated with better prognosis in patients with resected NSCLC (18). In addition to genomic alterations, the immune microenvironment also plays an important role in lung cancer. Tumor-infiltrating lymphocytes (TILs), reflecting the host immune response within the tumor microenvironment, have also been evaluated in this consortium and were associated with improved survival in patients with resected NSCLC (8).

Whether these tumor genomic and immune characteristics are associated with the risk of developing SPC remains unclear. Within the LACE-Bio consortium, detailed smoking variables are not consistently available across trials, which further motivates the exploration of tumor-derived biomarkers that may capture aspects of carcinogenic exposure. TMB in the primary tumor may partly reflect the cumulative effects of mutagenic exposures involved in lung carcinogenesis and may therefore provide indirect information on carcinogenic processes affecting the lung tissue. However, TMB measured in the primary tumor may not fully reflect the mutational processes occurring in other tissues at risk of developing an SPC. In addition, TIL infiltration reflects a local immune response within the tumor microenvironment. It may capture aspects of host antitumor immunity that could influence broader cancer surveillance mechanisms, although this relationship remains uncertain. Supporting this hypothesis, studies in other tumor types have suggested that higher levels of TILs may be associated with a lower incidence of SPCs (19).

The objective of this LACE-Bio study was, therefore, to investigate whether tumor genomic instability, assessed by TMB, and the host immune response, measured by the presence of TILs in primary resected tumors, were associated with the risk of developing SPC. We hypothesized that higher TMB and lower immune infiltration in the primary tumor would be associated with an increased risk of SPC.

## 2 Materials and methods

### 2.1 Study population

The LACE-Bio consortium includes patient data from three pivotal adjuvant randomized clinical trials evaluating postoperative platinum-based chemotherapy versus observation after complete resection of stage I to III NSCLC (the International Adjuvant Lung Trial [IALT] (20, 21), the Cancer and Leukemia Group B-9633 [CALGB] (22), and the Canadian Cancer Trials Group, previously the National Cancer Institute of Canada Clinical Trials Group [JBR.10] (23)). These trials established the role of adjuvant chemotherapy for completely resected NSCLC, and importantly, each study collected archival tissue specimens (formalin fixed, paraffin embedded [FFPE]) from consenting patients for future translational research studies. Each group maintains its own trial database and associated biobank. The LACE-Bio consortium facilitates validation of candidate biomarkers across multiple adjuvant NSCLC trials by harmonizing FFPE-based biomarker assays and evaluating their reproducibility and clinical associations in pooled and trial-stratified analyses.

### 2.2 Biomarkers

For the present study, TMB and TIL data were obtained from previously generated biomarker datasets within the LACE-Bio consortium, derived from archived FFPE tumor specimens collected from patients enrolled in the participating trials. TMB data used in this analysis were previously generated within the LACE-Bio consortium from 908 FFPE specimens from resected lung cancers using a custom targeted sequencing panel comprising 1, 538 genes selected based on The Cancer Genome Atlas (TCGA) Pan-Cancer analysis, designed to capture genes frequently mutated across multiple cancer types (10, 24). TMB estimated with this panel has been shown to correlate strongly with whole-exome sequencing-derived TMB in TCGA data, supporting its use for mutational burden estimation (18). Only nonsynonymous mutations were included in this analysis, as the functional consequences of synonymous mutations remain uncertain. TMB was classified into tertiles (low, moderate and high) using cut-offs derived from the distribution of TMB in the final analytic cohort. Additional details on sequencing procedures, variant calling, filtering strategies, and validation of the panel for TMB estimation are described in Devarakonda et al. (18).

TIL data were derived from prior LACE-Bio analyses in which hematoxylin and eosin-stained slides prepared from FFPE tumor specimens were reviewed independently by two pathologists (M-ST and EB) who were blinded to patient outcomes. TIL infiltration was initially evaluated using four categories (minimal, mild, moderate and intense) on hematoxylin and eosin-stained slides. For statistical analyses, these categories were grouped into a binary variable, where “marked” corresponds to intense lymphocytic infiltration (≥50% stromal lymphocytes) and “other” to minimal, mild, or moderate infiltration, as previously described (15). Discordant cases were reviewed jointly to reach a consensus classification.

### 2.3 Outcome

The primary endpoint was the occurrence of a second primary cancer (SPC). When a new lung tumor of identical histology was diagnosed, independent primary tumors were distinguished from recurrence according to the criteria proposed by Martini and Melamed (25) and Antakli et al. (26). Briefly, tumors of identical histology were considered independent primaries if they occurred at least 2 years apart, originated from carcinoma *in situ*, or arose in different lungs, lobes, or segments without common lymphatic involvement or distant metastases. Non-lung second primary cancers were ascertained by local investigators in each trial according to trial-specific procedures, and no central adjudication was performed.

### 2.4 Statistical analyses

Analyses were performed using a competing risks framework, with SPC and death without SPC considered as competing events. Follow-up was administratively censored at the last year in which SPCs were reported in each trial, as long-term follow-up for second cancers was not systematically collected by trial investigators.

Associations between TMB, TILs, and the competing endpoints were assessed using Fine and Gray subdistribution hazard models (27). Separate models were fitted for the SPC and death without SPC endpoints, treating the alternate event as a competing event. TMB and TILs were evaluated in distinct models. These models account for competing events by weighting individuals who remain at risk or who have experienced a competing event. Subdistribution hazard ratios (sHRs) and 95% confidence intervals (CI) were estimated for each category relative to a predefined reference group (sHR = 1). sHRs were interpreted as measures of the association between each biomarker and the cumulative incidence of the event of interest, accounting for the competing events.

All analyses were stratified by trial. Models were adjusted for treatment (Adjuvant chemotherapy vs. Observation) in unadjusted analyses, and additionally adjusted for age (≤50, 51-60, and >60), sex (Male vs. Female), tumor stage (T-stage; T1, T2, and T3/4), nodal stage (N-stage; N0, N1, and N2), histology (Squamous, Adenocarcinoma, and Other), WHO performance status (0 vs. ≥1), and surgery type (Pneumonectomy vs. Lobectomy/Other) in fully adjusted analyses. This prespecified adjustment strategy was applied consistently across previous LACE-Bio consortium analyses. For TMB, the moderate category was used as the reference group, and for TILs, the “Other” category was used as the reference group. Smoking variables were not consistently available across the trials and therefore were not included in the multivariable models.

In exploratory analyses, univariable and multivariable Fine and Gray subdistribution hazard models, including only clinical variables, were fitted to assess associations between clinicopathological characteristics and the cumulative incidence of SPC and death without SPC.

As sensitivity analyses, TMB was also modeled as a continuous variable. In addition, restricted cubic splines with three knots were used to explore potential non-linear relationships between TMB and the outcomes. Furthermore, to explore the potential impact of SPC heterogeneity, Fine and Gray competing risk models were repeated considering tobacco-related SPCs (lung, head and neck, bladder, and esophagus, according to the classification previously used within the LACE-Bio consortium) as the event of interest. Non-tobacco-related SPCs and deaths without SPC were treated as competing events. Finally, to evaluate the potential impact of heterogeneous trial-specific follow-up on SPC ascertainment, an additional sensitivity analysis was performed by administratively censoring follow-up at 5 years after randomization for all patients. The fully adjusted Fine and Gray models were then refitted using this common follow-up horizon. To assess the robustness of the fully adjusted models, given the limited number of SPC events, additional parsimonious sensitivity analyses were performed. These models remained stratified by trial and adjusted for treatment arm, but clinical adjustment was restricted to T-stage, N-stage and WHO performance status, selected *a priori* as key indicators of disease severity and prognosis.

Given the limited number of SPC events, we performed a *post hoc* detectable effect size assessment using a Schoenfeld-type approach (28) based on the observed number of events, assuming a two-sided α level of 0.05 and 80% power.

Statistical significance was set at p < 0.05. All statistical analyses were performed using R version 4.5.1, with the ‘survival’ package (version 3.8.3) and the ‘rms’ package (version 8.1.0).

## 3 Results

### 3.1 Sample availability, demographics and clinical characteristics

This study included 1, 013 patients from three LACE-Bio trials of whom 1, 010 had FFPE tumor blocks available for molecular assessment. Samples from 908 patients had sufficient tissue quantity and quality for targeted next-generation sequencing and successful TMB assessment. After exclusion of patients with missing TIL measurements (N = 22), or missing clinical covariates (N = 7), the final population comprised 879 patients (120 CALGB, 507 IALT and 252 JBR.10) (**Supplementary Figure 1**). Compared with the 879 included patients, the 29 excluded patients (3.2%) differed mainly with respect to trial, histological subtype and surgery type, whereas age, sex, treatment allocation and WHO performance status were broadly similar (**Supplementary Table 5**).

In the overall population, approximately half of the patients were aged 60 years or older, 73% were male, and about half had a WHO performance status of zero. Fewer than 15% of patients had stages T3/T4 disease, and the most frequent histological subtype was squamous cell carcinoma (**Table 1**).

**Table 1:** Baseline patient and primary tumor characteristics according to TMB and TILs.

Characteristic	TMB (mutations/MB)						TILs^a^				Total (N = 879)	
	Low (≤4) (N = 293)		Moderate (>4, ≤7.8) (N = 293)		High (>7.8) (N = 293)		Marked (N = 85)		Other (N = 794)			
	N	%^**^	N	%^**^	N	%^**^	N	%^**^	N	%^**^	N	%^***^
Age												
≤ 50	44	*27.33*	54	*33.54*	63	*39.13*	20	*12.42*	141	*87.58*	161	*18.32*
51-60	94	*33.33*	88	*31.21*	100	*35.46*	24	*8.51*	258	*91.49*	282	*32.08*
> 60	155	*35.55*	151	*34.63*	130	*29.82*	41	*9.40*	395	*90.60*	436	*49.60*
Sex												
Male	199	*31.09*	218	*34.06*	223	*34.84*	63	*9.84*	577	*90.16*	640	*72.81*
Female	94	*39.33*	75	*31.38*	70	*29.29*	22	*9.21*	217	*90.79*	239	*27.19*
T-stage*												
T1	42	*38.53*	38	*34.86*	29	*26.61*	21	*19.27*	88	*80.73*	109	*12.40*
T2	225	*34.30*	221	*33.69*	210	*32.01*	57	*8.69*	599	*91.31*	656	*74.63*
T3/T4	26	*22.81*	34	*29.82*	54	*47.37*	7	*6.14*	107	*93.86*	114	*12.97*
N-stage*												
N0	149	*32.75*	136	*29.89*	170	*37.36*	42	*9.23*	413	*90.77*	455	*51.76*
N1	101	*33.89*	111	*37.25*	86	*28.86*	36	*12.08*	262	*87.92*	298	*33.90*
N2	43	*34.13*	46	*36.51*	37	*29.36*	7	*5.56*	119	*94.44*	126	*14.33*
WHO performance status												
0	149	*33.26*	140	*31.25*	159	*35.49*	44	*9.82*	404	*90.18*	448	*50.97*
≥ 1	144	*33.41*	153	*35.50*	134	*31.09*	41	*9.51*	390	*90.49*	431	*49.03*
Histology												
Squamous	121	*29.51*	157	*38.29*	132	*32.20*	49	*11.95*	361	*88.05*	410	*46.64*
Adenocarcinoma	146	*40.00*	100	*27.40*	119	*32.60*	27	*7.40*	338	*92.60*	365	*41.52*
Other	26	*25.00*	36	*34.62*	42	*40.38*	9	*8.65*	95	*91.35*	104	*11.83*
Trials												
CALGB	66	*55.00*	29	*24.17*	25	*20.83*	5	*4.17*	115	*95.83*	120	*13.65*
IALT	134	*26.43*	169	*33.33*	204	*40.24*	60	*11.83*	447	*88.17*	507	*57.68*
JBR.10	93	*36.90*	95	*37.70*	64	*25.40*	20	*7.94*	232	*92.06*	252	*28.67*
Treatment												
Observation	152	*34.70*	138	*31.51*	148	*33.79*	41	*9.36*	397	*90.64*	438	*49.83*
Adjuvant chemotherapy	141	*31.97*	155	*35.15*	145	*32.88*	44	*9.98*	397	*90.02*	441	*50.17*
Surgery type												
Pneumonectomy	71	*25.44*	104	*37.28*	104	*37.28*	21	*7.53*	258	*92.47*	279	*31.74*
Lobectomy/Other	222	*37.00*	189	*31.50*	189	*31.50*	64	*10.67*	536	*89.33*	600	*68.26*

TMB, tumor mutational burden; TILs, tumor-infiltrating lymphocytes; WHO, World Health Organization.

^*^6th Edition TNM staging classification.

^**^Percentages are calculated across TMB categories and TIL categories within each row.

^***^Percentages are calculated using the overall study population as the denominator.

^a^TIL categories were defined as follows: “marked” = intense lymphocytic infiltration (≥50% stromal lymphocytes); “other” = minimal, mild, or moderate infiltration.

### 3.2 TMB results

In the overall population, TMB ranged from 0.19 to 225.36 mutations/megabase (MB), with a mean of 9.36 mutations/MB and a median of 5.68 mutations/MB (**Table 2**; **Supplementary Figure 2**). The distribution of TMB differed across the three trials (p = 1.10e-12), with higher TMB levels seen in tumor samples from the global IALT study compared to the two North American trials. TMB was categorized into tertiles corresponding to low (≤4 mutations/MB), moderate (>4 and ≤7.8 mutations/MB), and high (>7.8 mutations/MB). Among T1 tumors, 26.6% were classified in the high TMB group, compared with 32.0% of T2 tumors and 47.4% of T3/T4 tumors. High TMB was observed in 32% of squamous cancers compared to 34% of other histologic subtypes (**Table 1**).

**Table 2:** Distribution of TMB and TILs in primary lung tumors according to trial in the overall study population and among patients who developed a second primary cancer.

Overall study population	Total	CALGB	IALT	JBR.10	P value
	(N = 879)	(N = 120)	(N = 507)	(N = 252)	
TMB					
Mean [Min; Max]	9.36 [0.19; 225.36]	4.94 [0.19; 21.69]	11.49 [0.28; 225.36]	7.19 [0.30; 162.69]	
Median [Q1; Q3]	5.68 [3.24; 9.70]	3.53 [2.21; 6.77]	6.64 [3.84; 12.02]	4.87 [2.89; 7.82]	1.10e-12
**TMB (3-level) (%)**					1.38e-09
Low	293 (33.33%)	66 (55.00%)	134 (26.43%)	93 (36.90%)	
Moderate	293 (33.33%)	29 (24.17%)	169 (33.33%)	95 (37.70%)	
High	293 (33.33%)	25 (20.83%)	204 (40.24%)	64 (25.40%)	
TILs^a^					0.02
Marked	85 (9.67%)	5 (4.17%)	60 (11.83%)	20 (7.94%)	
Other	794 (90.33%)	115 (95.83%)	447 (88.17%)	232 (92.06%)	
Patients who developed SPC	(N = 47)	(N = 6)	(N = 19)	(N = 22)	
TMB					
Mean [Min; Max]	9.79 [0.30; 90.09]	7.62 [2.97; 12.28]	14.06 [1.54; 90.09]	6.69 [0.30; 23.64]	
Median [Q1; Q3]	5.58 [3.12; 8.87]	7.36 [4.26; 11.23]	5.43 [2.88; 7.91]	6.23 [3.26; 8.09]	0.67
**TMB (3-level) (%)**					0.73
Low	15 (31.91%)	1 (16.67%)	8 (42.10%)	6 (27.27%)	
Moderate	17 (36.17%)	2 (33.33%)	6 (31.58%)	9 (40.91%)	
High	15 (31.91%)	3 (50.00%)	5 (26.32%)	7 (31.82%)	
**TILs^a^**					0.06
Marked	4 (8.51%)	0 (0.00%)	4 (21.05%)	0 (0.00%)	
Other	43 (91.49%)	6 (100.00%)	15 (78.95%)	22 (100.00%)	

TMB, tumor mutational burden; TILs, tumor-infiltrating lymphocytes; SPC, second primary cancer; Q1, 1^st^ quartile; Q3, 3^rd^ quartile.

^*^P values were obtained using the Kruskal-Wallis test for continuous variables and the chi-square test or Fisher’s exact test, as appropriate, for categorical variables.

^a^TIL categories were defined as follows: “marked” = intense lymphocytic infiltration (≥50% stromal lymphocytes); “other” = minimal, mild, or moderate infiltration.

Bold values indicate statistical significance at p < 0.05.

### 3.3 TILs results

Only 85 tumor samples had marked TILs. Marked TIL infiltration was less frequent in the adenocarcinoma group (7.4%) than in squamous cell carcinoma (12%) or other histologic subtypes (8.7%). Marked TIL infiltration was observed in only 6.1% of stage T3/T4 cancers compared to 10.2% of stages T1 and T2 cancers combined (**Table 1**). TILs’ distribution varied across trials, with more observed marked TILs in the IALT trial (p = 0.02, **Table 2**). Across trials, tumors with marked TIL infiltration tended to display higher and more heterogeneous TMB values compared with tumors classified as having “Other” TIL patterns, although substantial between-trial heterogeneity was observed (**Supplementary Figure 2**).

### 3.4 Second primary cancers and competing events

In our study population, 47 patients developed a second primary cancer (SPC) (**Table 2**), including 22 cases from JBR.10 (last observed case in 2004), 19 cases in IALT (last observed case in 2002) and 6 cases in CALGB (last observed case in 2008). Among these, 15 (31.9%) were second primary lung cancers. Other SPCs included head and neck (n = 6), prostate (n = 5), breast (n = 3), colon and rectum (n = 3), bladder (n = 2), esophagus (n = 1), stomach (n = 1), Hodgkin disease (n = 1), and cancers classified as “Other” (n = 10) (**Supplementary Table 3**). The “Other” category includes SPCs arising from localization sites not represented in the predefined LACE-Bio classification (lung, bladder, head and neck, colon and rectum, breast, prostate, esophagus, stomach, pancreas, cervix, small bowel, and Hodgkin disease). According to the tobacco-related cancer classification previously used within the LACE-Bio consortium (lung, bladder, head and neck, and esophagus), 24 of the 47 SPCs (51.1%) were classified as tobacco-related malignancies (**Supplementary Table 3**).

Among patients who developed an SPC, TMB ranged from 0.30 to 90.09 mutations/MB, with a mean of 9.79 mutations/MB and a median of 5.58 mutations/MB. The rate of SPCs in the three TMB tertiles was similar, with 31%, 36% and 31% in low, moderate and high TMB groups, respectively (**Table 2**). SPCs were observed in 4 (8.51%) patients with marked TILs and 43 (91.49%) patients with “Other” TIL infiltration (**Table 2**).

For the competing event, a total of 392 deaths without developing SPC were observed (52 in CALGB, 251 in IALT, and 89 in JBR.10). Given the limited number of SPC events (n = 47), the power to detect modest associations was limited. The study had approximately 80% power to detect only relatively large effect sizes for SPC, with detectable subdistribution hazard ratios of about 2.2 or greater for TMB comparisons between similarly sized groups, and substantially larger effect sizes for TIL analyses because of the small proportion of patients with marked TILs.

### 3.5 Survival analyses

At the time of analysis, the median follow-up estimated using the reverse Kaplan-Meier method was 7.35 years ([95% CI, 6.76-7.92]), 4.76 years ([95% CI, 4.56-5.05]), and 5.48 years ([95% CI, 5.12-6.09]) in the CALGB, IALT, and JBR.10 trials, respectively.

We first examined univariable associations between clinical factors and the cumulative incidence of SPC. The cumulative incidence of SPC increased with advancing age, with higher subdistribution hazards observed in patients aged 51–60 years (sHR = 2.73 [95% CI, 0.78-9.55]) and >60 years (sHR = 3.75 [95% CI, 1.16-12.10]) compared to those aged ≤50 years (global p value = 0.03). No significant difference in the cumulative incidence of SPC was observed between men and women (global p = 0.36). Patients with squamous cell carcinoma had a higher cumulative incidence of SPC than those with adenocarcinoma (sHR = 2.00 [95% CI, 1.07-3.73], p = 0.03). There was no significant difference in the cumulative incidence of SPC between the adjuvant chemotherapy and observation arm (sHR = 1.32 [95% CI, 0.75-2.31], p = 0.34) (**Table 3**). Unfortunately, the effect of prior or current smoking status could not be assessed, as this variable was not collected in IALT, the largest of the LACE-Bio trials.

**Table 3:** Association between clinicopathological variables and competing endpoints (univariable Fine and Gray models).

Characteristic	SPC (E = 47)			Death without SPC (E = 392)		
	sHR (95% CI)	P value	Global P value^**^	sHR (95% CI)	P value	Global P value^**^
Age						
≤ 50 (ref)	1.00		**0.03**	1.00		0.18
51-60	2.73 (0.78-9.55)	0.12		1.17 (0.86-1.60)	0.32	
>60	3.75 (1.16-12.10)	0.03		1.30 (0.97-1.74)	0.08	
Sex						
Female (ref)	1.00		0.36	1.00		**1.26e-04**
Male	1.37 (0.69-2.70)	0.37		1.60 (1.25-2.04)	2.39e-04	
T-stage*						
T1 (ref)	1.00		0.11	1.00		**5.32e-05**
T2	0.43 (0.21-0.86)	0.02		1.35 (0.98-1.87)	0.07	
T3/4	0.48 (0.14-1.58)	0.23		2.29 (1.59-3.30)	9.00e-06	
N-stage*						
N0 (ref)	1.00		1.00	1.00		**6.20e-14**
N1	0.99 (0.52-1.88)	0.97		1.64 (1.29-2.10)	7.06e-05	
N2	1.04 (0.36-2.95)	0.95		3.21 (2.43-4.25)	3.21e-16	
WHO performance status						
0 (ref)	1.00		0.75	1.00		**0.01**
≥ 1	0.91 (0.51-1.61)	0.75		1.29 (1.06-1.58)	0.01	
Histology						
Adenocarcinoma (ref)	1.00		**0.03**	1.00		0.26
Squamous	2.00 (1.07-3.73)	0.03		0.95 (0.76-1.17)	0.61	
Other	0.69 (0.20-2.41)	0.56		1.23 (0.89-1.70)	0.20	
Surgery type						
Lobectomy/Other (ref)	1.00		0.22	1.00		**8.10e-04**
Pneumonectomy	0.65 (0.32-1.31)	0.23		1.44 (1.17-1.77)	5.79e-04	
Treatment						
Observation (ref)	1.00		0.35	1.00		0.21
Adjuvant chemotherapy	1.32 (0.75-2.31)	0.34		0.88 (0.72-1.07)	0.21	

E, events; SPC, second primary cancer; sHR, subdistribution hazard ratio; WHO, World Health Organization; ref, reference group; CI, confidence interval.

^*^6th Edition TNM staging classification.

^**^Global P values correspond to likelihood ratio tests.

Bold values indicate statistical significance at p < 0.05.

For the competing endpoint of death without developing SPC, men had a higher cumulative incidence of death compared to women (sHR = 1.60 [95% CI, 1.25-2.04], p = 1.26e-04). No significant association between the cumulative incidence of death without SPC and age was observed (global p value = 0.18). Death without SPC was associated with T-stage and N-stage, with higher subdistribution hazards observed for more advanced stages. WHO performance status and type of surgery were also associated with this endpoint (**Table 3**). Results from multivariable analyses adjusting for all clinical covariates were generally consistent in direction with those observed in univariable analyses. However, associations with WHO performance status and surgery type were no longer statistically significant after adjustment (p = 0.14, **Supplementary Table 2**).

Neither univariable nor multivariable analyses showed a significant association between TIL categories and SPC (univariable sHR = 0.98 [95% CI, 0.36-2.69], p = 0.97; multivariable sHR = 0.83 [95% CI, 0.32-2.19], p = 0.71) (**Tables 4**, **5**). The lack of association may be related to the small number of patients with marked TILs among those who developed SPC (N = 4). For the competing endpoint of death without SPC, univariable analysis showed a lower cumulative incidence of death among patients with marked TILs (sHR = 0.61 [95% CI, 0.41-0.90], p = 0.01). This association was attenuated after adjustment for clinical covariates (sHR = 0.71 [95% CI, 0.46-1.10], p = 0.12) (**Tables 4**, **5**). Consistently, Aalen-Johansen estimates of the cumulative incidence of death without SPC showed a lower probability of death in patients with marked TILs compared with the “other” TILs category (**Figure 1B**).

**Table 4:** Association between TMB, TILs, and competing endpoints (unadjusted Fine and Gray models).

Biomarker	TMB			TILs		
	sHR^*^ (95% CI)	P value	Global P value^**^	sHR^*^ (95% CI)	P value	Global P value^**^
SPC (E = 47)						
** TILs^a^**						0.97
Marked (E = 4)				0.98 (0.36-2.69)	0.97	
Other (ref) (E = 43)				1.00		
TMB			0.90			
Low (E = 15)	0.87 (0.42-1.79)	0.71				
Moderate (ref) (E = 17)	1.00					
High (E = 15)	0.87 (0.44-1.71)	0.68				
TMB (Continuous)	1.00 (0.99-1.02)	0.80	0.82			
Spline (test for non-linearity)^b^		0.81				
Death without SPC (E = 392)						
** TILs^a^**						**0.01**
Marked (E = 28)				0.61 (0.41-0.90)	**0.01**	
Other (ref) (E = 364)				1.00		
** TMB**			0.11			
Low (E = 147)	1.35 (1.06-1.72)	**0.01**				
Moderate (ref) (E = 119)	1.00					
High (E = 126)	0.96 (0.74-1.24)	0.75				
TMB (Continuous)	0.99 (0.99-1.00)	0.22	0.15			
Spline (test for non-linearity)^b^		0.10				

TMB, tumor mutational burden; TILs, tumor-infiltrating lymphocytes; E, events; SPC, second primary cancer; sHR, subdistribution hazard ratio; ref, reference group; CI, confidence interval.

^*^Fine and Gray models were stratified by trial and adjusted for treatment only.

^**^Global P values correspond to likelihood ratio tests.

^a^TIL categories were defined as follows: “marked” = intense lymphocytic infiltration (≥50% stromal lymphocytes); “other” = minimal, mild, or moderate infiltration.

^b^Non-linearity was assessed using restricted cubic spline models with three knots.

Bold values indicate statistical significance at p < 0.05.

**Table 5:** Association between TMB, TILs, and competing endpoints (fully adjusted Fine and Gray models).

Biomarker	TMB			TILs		
	sHR^*^ (95% CI)	P value	Global P value^**^	sHR^*^ (95% CI)	P value	Global P value^**^
SPC (E = 47)						
** TILs^a^**						0.73
Marked (E = 4)				0.83 (0.32-2.19)	0.71	
Other (ref) (E = 43)				1.00		
TMB			0.88			
Low (E = 15)	0.83 (0.40-1.75)	0.63				
Moderate (ref) (E = 17)	1.00					
High (E = 15)	0.91 (0.46-1.83)	0.80				
TMB (Continuous)	1.00 (0.99-1.02)	0.60	0.64			
Spline (test for non-linearity)^b^		0.95				
Death without SPC (E = 392)						
** TILs^a^**						0.08
Marked (E = 28)				0.71 (0.46-1.10)	0.12	
Other (ref) (E = 364)				1.00		
** TMB**			**0.03**			
Low (E = 147)	1.35 (1.06-1.72)	**0.02**				
Moderate (ref) (E = 119)	1.00					
High (E = 126)	1.03 (0.79-1.33)	0.84				
TMB (Continuous)	1.00 (0.99-1.00)	0.25	0.23			
Spline (test for non-linearity)^b^		0.17				

TMB, tumor mutational burden; TILs, tumor-infiltrating lymphocytes; E, events; SPC, second primary cancer; sHR, subdistribution hazard ratio; ref, reference group; CI, confidence interval.

^*^Fine and Gray models were stratified by trial, and adjusted for all clinical characteristics (treatment, age, sex, tumor stage, nodal stage, histology, WHO performance status and surgery type).

^**^Global P values correspond to likelihood ratio tests.

^a^TIL categories were defined as follows: “marked” = intense lymphocytic infiltration (≥50% stromal lymphocytes); “other” = minimal, mild, or moderate infiltration.

^b^Non-linearity was assessed using restricted cubic spline models with three knots.

Bold values indicate statistical significance at p < 0.05.

**Figure 1:**
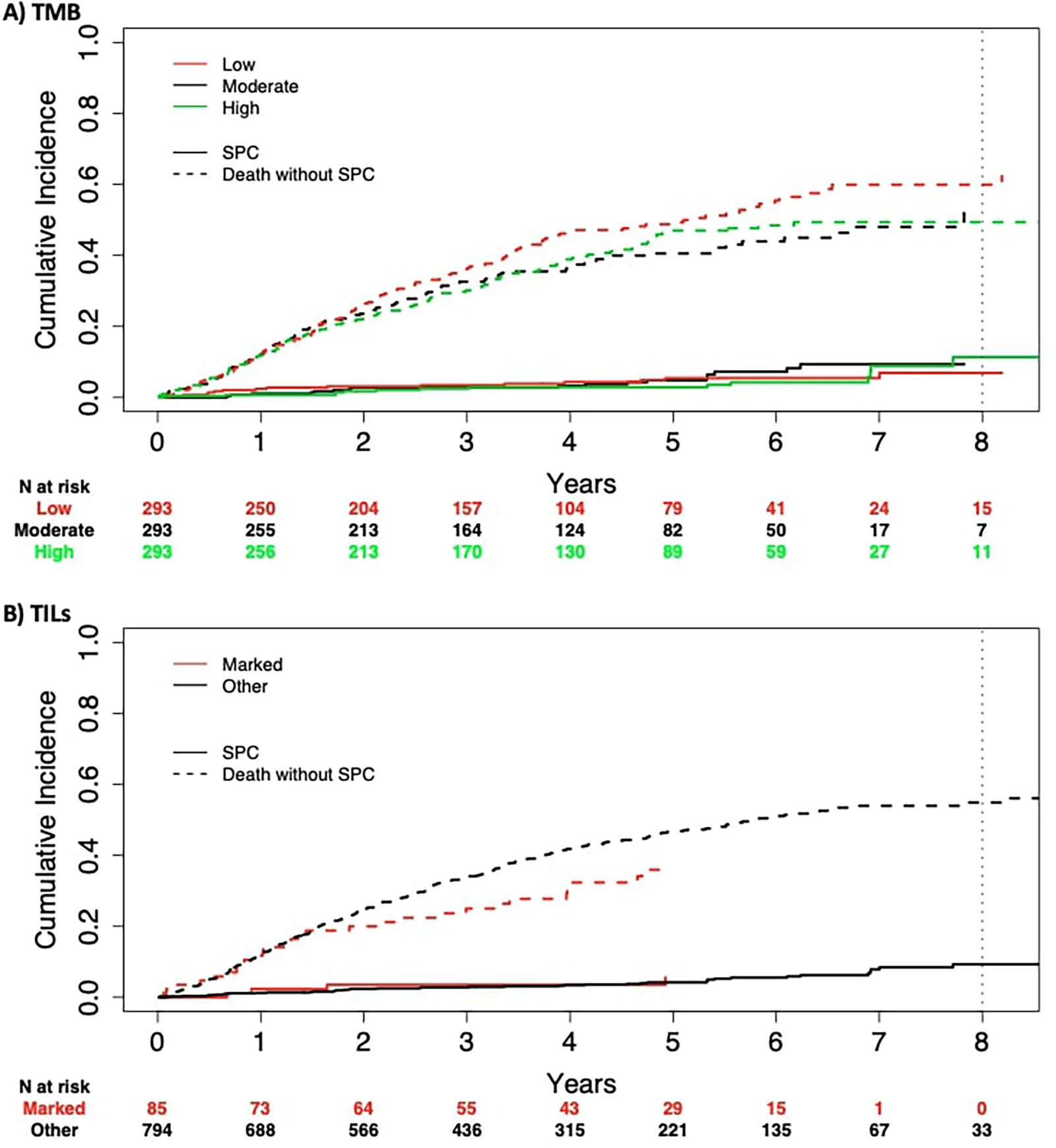
Aalen-Johansen estimates of cumulative incidence functions for SPC (solid lines) and death without developing SPC (dashed lines) **(A)** according to TMB categories (Low [red], moderate [black], and high [green]) **(B)** according to TILs categories (Marked [red] vs other [black]). SPC, second primary cancer; TMB, tumor mutational burden; TILs, tumor-infiltrating lymphocytes. TIL categories were defined as follows: “marked” = intense lymphocytic infiltration (≥50% stromal lymphocytes); “other” = minimal, mild, or moderate infiltration.

For TMB, no clear association was observed between TMB categories and the cumulative incidence of SPC (**Tables 4**, **5**). In contrast, a significant association was observed between low TMB and the competing endpoint of death without developing SPC (univariable sHR = 1.35 [95% CI, 1.06-1.72], p = 0.01 and multivariable sHR = 1.35 [95% CI, 1.06-1.72], p = 0.02). Consistently, Aalen-Johansen estimates of the cumulative incidence of death without SPC showed a higher probability of death among patients with low TMB compared with those with moderate or high TMB (**Figure 1A**). When TMB was modeled as a continuous variable, no significant association with SPC risk was observed (sHR = 1.00 [95% CI, 0.99-1.02]) (**Table 5**). In analyses using restricted cubic splines with three knots, there was no clear evidence of a non-linear association between TMB and SPC risk (p for non-linearity = 0.95) (**Supplementary Figure 3**). Similar results were observed for the competing endpoint of death without SPC, with no clear evidence of a non-linear association.

In sensitivity analysis, we repeated the competing risk analyses considering tobacco-related SPCs as the event of interest. The estimated associations between TMB and tobacco-related SPCs were broadly consistent with those observed for the overall SPC endpoint. Additional parsimonious Fine and Gray models stratified by trial and adjusted only for treatment arm, T-stage, N-stage, and WHO performance status yielded estimates and conclusions consistent with those of the prespecified fully adjusted models (**Supplementary Table 4**). Owing to the very limited number of tobacco-related SPCs occurring among patients with marked TILs, subgroup analyses for TILs were considered unreliable and are not reported.

The sensitivity analysis with administrative censoring of follow-up at 5 years after randomization yielded conclusions consistent with those of the primary analyses. No association was observed between TMB or TILs and SPC, whereas the association between low TMB and death without SPC remained of similar magnitude (**Supplementary Table 6**).

## 4 Discussion

Previous analyses from the LACE-Bio group reported that high nonsynonymous TMB and intense tumor-infiltrating lymphocytes were associated with improved survival in patients with resected NSCLC (15, 18). The association between tumor cells and lymphocytes has led to the hypothesis that adaptive immunity may contribute to maintaining occult cancer in a quiescent state. Experimental models, inferred from a mouse model (29), suggest that immune surveillance can shape tumor evolution and delay malignant progression (30). In addition, tumor mutational burden has been shown to correlate with tobacco exposure, with higher mutation burdens observed in smokers compared with nonsmokers across several tobacco-related cancers (31). Because smoking is a major risk factor for the development of second primary cancers following curative resection of NSCLC (4), TMB may partly reflect carcinogenic processes that contribute to SPC risk. Taken together, these observations provided the rationale for investigating whether tumor genomic and immune characteristics of the primary tumor might also influence the development of second primary cancers (SPC).

Few studies have focused on SPCs, and most have primarily reported incidence rates, tumor characteristics, outcomes, and treatment effectiveness. To our knowledge, none has evaluated the potential role of tumor genomic or immune-related biomarkers in the development of SPC. The present study, therefore, aimed to investigate the potential contributions of TMB and TILs to the occurrence of SPC in patients with resected NSCLC.

Contrary to our initial hypothesis, we did not detect a statistically significant association between TMB or TILs and the cumulative incidence of SPC. No clear trend was identified across TMB categories, and marked TILs was similarly not associated with SPC occurrence. In contrast, we did identify an association between TMB and the competing endpoint of death without SPC. Patients with low TMB experienced a higher cumulative incidence of death without developing SPC compared with those with moderate TMB, whereas no statistically significant difference was observed for the high TMB group. This finding is consistent with the literature showing that high nonsynonymous TMB is associated with improved survival in patients with resected NSCLC (18). In this clinical context, the evaluation of SPC is strongly influenced by competing events, including cancer recurrence and death from cancer or other causes. Patients with higher TMB may survive longer after curative treatment and therefore remain at risk of developing an SPC, whereas patients with lower TMB may die earlier and thus have less opportunity for SPC to occur. Death from non-cancer causes is frequent in lung cancer patients because of the high prevalence of smoking-related comorbidities. Therefore, patients must survive long enough after curative treatment for an SPC to be observed, and differences in survival may substantially affect the probability of detecting SPC.

One possible explanation for the absence of association between TMB or TILs and SPC is the relatively small number of SPC events observed in the LACE-Bio data, as only 47 SPC events were observed. This limited number of events reduced the statistical power to detect modest associations between the investigated biomarkers and SPC risk. A *post hoc* detectable effect size assessment suggested that, given the 47 SPC events, the study was primarily powered to detect relatively large associations (subdistribution hazard ratios of approximately two-fold or greater). Consequently, modest but potentially clinically meaningful effects of TMB or TILs on SPC risk may have remained undetected. This limitation was particularly relevant for TIL analyses, given the small number of tumors with marked lymphocytic infiltration and the very small number of SPC events observed in this subgroup.

The limited number of SPC events prevented meaningful analyses of individual SPC types, necessitating a composite SPC endpoint. However, this endpoint encompassed cancers arising from diverse sites with potentially distinct biological mechanisms. Although 15 of the 47 SPCs (31.9%) were second primary lung cancers and 24 (51.1%) were classified as tobacco-related malignancies according to the predefined LACE-Bio classification, the remaining SPCs arose from diverse sites with potentially distinct etiologies. Consequently, associations specific to SPC subtypes may have been diluted when all SPCs were analyzed together. To further explore this possibility, we performed an additional sensitivity analysis restricted to tobacco-related SPCs. The estimated associations for TMB were broadly similar to those observed for the overall SPC endpoint, although these analyses were limited by the small number of events and should therefore be interpreted with caution. On the other hand, the analysis of TILs was based on only four SPC events among patients with marked TILs, all of which occurred in the IALT trial, whereas no marked TILs-SPC events were observed in CALGB or JBR.10. This trial-specific sparsity limited the precision of the estimated association between TILs and SPC risk. To further assess the robustness of these findings, given the limited number of SPC events, we conducted additional parsimonious sensitivity analyses using a reduced set of prespecified clinical adjustment variables. These analyses yielded results consistent with those obtained from the prespecified fully adjusted models.

Another limitation relates to the ascertainment of SPCs in the original trials. Long-term follow-up for SPC was not systematically captured and therefore required administrative censoring at the last year in which SPCs were reported for each trial. As a result, the effective follow-up for the SPC endpoint differed across trials, and later SPC events may have been under-ascertained. Although our analyses were stratified by trial to account for differences between studies, this approach cannot fully address potential under-detection of SPC occurring after the end of trial-specific follow-up. To evaluate the potential impact of this limitation, we performed a sensitivity analysis administratively censoring follow-up at 5 years after randomization to provide a common follow-up horizon across all trials. The conclusions remained consistent with those of the primary analyses, suggesting that heterogeneous follow-up across trials had little impact on the overall conclusions.

Finally, smoking is a major risk factor for SPCs and is also associated with TMB. Because smoking variables were not consistently collected across the included trials, residual confounding related to tobacco exposure cannot be excluded.

These limitations highlight the need for further studies to better characterize factors associated with SPC development. Given the relatively low incidence of SPC, larger cohorts with longer follow-up, particularly in patients with earlier-stage disease, will be required to better investigate potential biomarkers associated with SPC risk. Future studies may also benefit from exploring the heterogeneity of SPC by examining specific cancer types, such as second primary lung cancers, which may arise through distinct etiological mechanisms compared with other malignancies. In addition to traditional tumor-based biomarkers, emerging approaches such as circulating tumor DNA (ctDNA) are increasingly being explored in early-stage NSCLC. However, their potential role in predicting the risk of second primary cancers remains unclear and warrants further investigation.

## 5 Conclusion

This study represents the first evaluation of tumor genomic instability, assessed by TMB, and host immune response, assessed by TILs, in relation to competing events of SPC and death without SPC in patients with completely resected NSCLC. Overall, our findings did not show a clear association between TMB or TILs and the cumulative incidence of SPC, whereas low TMB (relative to the moderate TMB group) was associated with the competing endpoint of death without developing SPC. These results highlight the importance of accounting for competing risks when evaluating long-term outcomes such as SPC. However, the number of SPC events was limited, and modest associations cannot be excluded. Larger studies with longer follow-up will therefore be required to further investigate potential biomarkers of SPC risk and to validate these findings in independent cohorts of patients with early-stage lung cancer.

## Data Availability

Access to the data analyzed in this study is subject to approval by the LACE-Bio consortium. Requests to access these datasets should be directed to the corresponding author.
