# Supplementary material for: Association of tumor mutational burden and tumor-infiltrating lymphocytes with second primary cancer after complete resection of non-small cell lung cancer
Source: Front Oncol. 2026 Sep 17;16:1773288. doi: 10.3389/fonc.2026.1773288 (PMC13626909; doi:10.3389/fonc.2026.1773288)
Supplement: Supplementary file 1 [file SupplementaryFile1.docx]

Supplementary Material

# 1 Supplementary Figures and Tables

## 1.1 Supplementary Tables

Supplementary Table 1. Baseline patient and primary tumor characteristics according to TMB and TILs among patients who developed a second primary cancer

|  | **TMB (mutations/MB)** | | | | | | **TILs^a^** | | | | **Total**  **(N = 47)** | |
| --- | --- | --- | --- | --- | --- | --- | --- | --- | --- | --- | --- | --- |
|  | **Low (≤4)**  **(N = 15)** | | **Moderate (>4, ≤7.8)**  **(N = 17)** | | **High (>7.8)**  **(N = 15)** | | **Marked**  **(N = 4)** | | **Other**  **(N = 43)** | |  |  |
|  | **N** | ***%^**^*** | **N** | ***%^**^*** | **N** | ***%^**^*** | **N** | ***%^**^*** | **N** | ***%^**^*** | **N** | ***%^***^*** |
| **Age** |  |  |  |  |  |  |  |  |  |  |  |  |
| ≤ 50 | 1 | *33.33* | 1 | *33.33* | 1 | *33.33* | 0 | *0.00* | 3 | *100.00* | 3 | *6.38* |
| 51-60 | 5 | *38.46* | 3 | *23.08* | 5 | *38.46* | 1 | *7.69* | 12 | *92.31* | 13 | *27.66* |
| > 60 | 9 | *29.03* | 13 | *41.94* | 9 | *29.03* | 3 | *9.68* | 28 | *90.32* | 31 | *65.96* |
| **Sex** |  |  |  |  |  |  |  |  |  |  |  |  |
| Male | 14 | *38.89* | 15 | *41.67* | 7 | *19.44* | 4 | *11.11* | 32 | *88.89* | 36 | *76.60* |
| Female | 1 | *9.09* | 2 | *18.18* | 8 | *72.73* | 0 | *0.00* | 11 | *100.00* | 11 | *23.40* |
| **T-stage*** |  |  |  |  |  |  |  |  |  |  |  |  |
| T1 | 4 | *40.00* | 3 | *30.00* | 3 | *30.00* | 4 | *40.00* | 6 | *60.00* | 10 | *21.28* |
| T2 | 9 | *27.27* | 13 | *39.39* | 11 | *33.33* | 0 | *0.00* | 33 | *100.00* | 33 | *70.21* |
| T3/T4 | 2 | *50.00* | 1 | *25.00* | 1 | *25.00* | 0 | *0.00* | 4 | *100.00* | 4 | *8.51* |
| **N-stage*** |  |  |  |  |  |  |  |  |  |  |  |  |
| N0 | 5 | *20.00* | 10 | *40.00* | 10 | *40.00* | 2 | *8.00* | 23 | *92.00* | 25 | *53.19* |
| N1 | 8 | *47.06* | 5 | *29.41* | 4 | *23.53* | 2 | *11.76* | 15 | *88.24* | 17 | *36.17* |
| N2 | 2 | *40.00* | 2 | *40.00* | 1 | *20.00* | 0 | *0.00* | 5 | *100.00* | 5 | *10.64* |
| **WHO performance status** |  |  |  |  |  |  |  |  |  |  |  |  |
| 0 | 8 | *32.00* | 9 | *36.00* | 8 | *32.00* | 1 | *4.00* | 24 | *96.00* | 25 | *53.19* |
| ≥ 1 | 7 | *31.82* | 8 | *36.36* | 7 | *31.82* | 3 | *13.64* | 19 | *86.36* | 22 | *46.81* |
| **Histology** |  |  |  |  |  |  |  |  |  |  |  |  |
| Squamous | 11 | *37.93* | 11 | *37.93* | 7 | *24.14* | 3 | *10.34* | 26 | *89.66* | 29 | *61.70* |
| Adenocarcinoma | 3 | *20.00* | 5 | *33.33* | 7 | *46.67* | 1 | *6.67* | 14 | *93.33* | 15 | *31.92* |
| Other | 1 | *33.33* | 1 | *33.33* | 1 | *33.33* | 0 | *0.00* | 3 | *100.00* | 3 | *6.38* |
| **Trials** |  |  |  |  |  |  |  |  |  |  |  |  |
| CALGB | 1 | *16.67* | 2 | *33.33* | 3 | *50.00* | 0 | *0.00* | 6 | *100.00* | 6 | *12.77* |
| IALT | 8 | *42.11* | 6 | *31.58* | 5 | *26.32* | 4 | *21.05* | 15 | *78.95* | 19 | *40.43* |
| JBR.10 | 6 | *27.27* | 9 | *40.91* | 7 | *31.82* | 0 | *0.00* | 22 | *100.00* | 22 | *46.81* |
| **Treatment** |  |  |  |  |  |  |  |  |  |  |  |  |
| Observation | 8 | *40.00* | 5 | *25.00* | 7 | *35.00* | 1 | *5.00* | 19 | *95.00* | 20 | *42.55* |
| Adjuvant chemotherapy | 7 | *25.93* | 12 | *44.44* | 8 | *29.63* | 3 | *11.11* | 24 | *88.89* | 27 | *57.45* |
| **Surgery type** |  |  |  |  |  |  |  |  |  |  |  |  |
| Pneumonectomy | 6 | *60.00* | 1 | *10.00* | 3 | *30.00* | 1 | *10.00* | 9 | *90.00* | 10 | *31.74* |
| Lobectomy/Other | 9 | *24.32* | 16 | *43.24* | 12 |  | 3 | *8.11* | 34 | *91.89* | 37 | *68.26* |

TMB, tumor mutational burden; TILs, tumor-infiltrating lymphocytes; WHO, World Health Organization; SPC, second primary cancer.
^*^ 6th Edition TNM staging classification.
^**^ Percentages are calculated across TMB categories and TIL categories within each row.
^***^ Percentages are calculated using the SPC population as the denominator.
^a^ TIL categories were defined as follows: “marked” = intense lymphocytic infiltration (≥50% stromal lymphocytes); “other” = minimal, mild, or moderate infiltration.

Supplementary Table 2. Association between clinicopathological variables and competing endpoints (multivariable Fine and Gray models)

|  | **SPC (E = 47)** | | | **Death without SPC (E = 392)** | | |
| --- | --- | --- | --- | --- | --- | --- |
|  | **sHR (95% CI)** | **P value** | **Global P value^**^** | **sHR (95% CI)** | **P value** | **Global P value^**^** |
| **Age** |  |  |  |  |  |  |
| ≤ 50 (ref) | 1.00 |  | 0.12 | 1.00 |  | 0.14 |
| 51-60 | 2.17 (0.61-7.78) | 0.23 |  | 1.12 (0.82-1.54) | 0.48 |  |
| >60 | 2.97 (0.94-9.39) | 0.06 |  | 1.31 (0.97-1.76) | 0.08 |  |
| **Sex** |  |  |  |  |  |  |
| Female (ref) | 1.00 |  | 0.80 | 1.00 |  | **2.99e-05** |
| Male | 1.10 (0.54-2.22) | 0.80 |  | 1.72 (1.33-2.22) | 2.74e-05 |  |
| **T-stage*** |  |  |  |  |  |  |
| T1 (ref) | 1.00 |  | 0.12 | 1.00 |  | **1.14e-04** |
| T2 | 0.42 (0.20-0.88) | 0.02 |  | 1.34 (0.96-1.86) | 0.08 |  |
| T3/4 | 0.50 (0.14-1.70) | 0.26 |  | 2.36 (1.56-3.57) | 4.89e-05 |  |
| **N-stage*** |  |  |  |  |  |  |
| N0 (ref) | 1.00 |  | 0.79 | 1.00 |  | **4.63e-14** |
| N1 | 0.98 (0.50-1.93) | 0.96 |  | 1.74 (1.34-2.25) | 2.87e-05 |  |
| N2 | 1.45 (0.48-4.39) | 0.51 |  | 3.50 (2.63-4.64) | <2.00e-16 |  |
| **WHO performance status** |  |  |  |  |  |  |
| 0 (ref) | 1.00 |  | 0.56 | 1.00 |  | 0.14 |
| ≥ 1 | 0.84 (0.47-1.50) | 0.55 |  | 1.16 (0.96-1.41) | 0.13 |  |
| **Histology** |  |  |  |  |  |  |
| Adenocarcinoma (ref) | 1.00 |  | **0.03** | 1.00 |  | **6.94e-05** |
| Squamous | 2.15 (1.11-4.16) | 0.02 |  | 0.61 (0.48-0.78) | 1.07e-04 |  |
| Other | 0.76 (0.22-2.65) | 0.67 |  | 1.07 (0.78-1.46) | 0.69 |  |
| **Surgery type** |  |  |  |  |  |  |
| Lobectomy/Other (ref) | 1.00 |  | 0.13 | 1.00 |  | 0.89 |
| Pneumonectomy | 0.54 (0.24-1.21) | 0.13 |  | 1.02 (0.79-1.31) | 0.89 |  |
| **Treatment** |  |  |  |  |  |  |
| Observation (ref) | 1.00 |  | 0.34 | 1.00 |  | 0.48 |
| Adjuvant chemotherapy | 1.33 (0.75-2.36) | 0.33 |  | 0.93 (0.76-1.13) | 0.47 |  |

E, events; SPC, second primary cancer; sHR, subdistribution hazard ratio; WHO, World Health Organization; ref, reference group; CI, confidence interval. ^*^ 6th Edition TNM staging classification ^**^ Global P values correspond to likelihood ratio tests.

Supplementary Table 3. Characteristics of second primary cancers according to localization site

| **Characteristic** | **Tobacco-related SPCs (n = 24)** | | | | |  | **Non-tobacco-related SPCs (n = 23)** | | | | | | | **Overall**  **(n = 47)** |
| --- | --- | --- | --- | --- | --- | --- | --- | --- | --- | --- | --- | --- | --- | --- |
|  | **Lung**  **(n = 15)** | **Bladder**  **(n = 2)** | **Head and neck**  **(n = 6)** | **Esophagus**  **(n = 1)** | **Total** |  | **Breast**  **(n = 3)** | **Prostate**  **(n = 5)** | **Colon and rectum**  **(n = 3)** | **Stomach**  **(n = 1)** | **Hodgkin disease**  **(n = 1)** | **Other^*^**  **(n = 10)** | **Total** |  |
| **Time to SPC (years)** |  |  |  |  |  |  |  |  |  |  |  |  |  |  |
| Median [Q1; Q3] |  |  |  |  | 4.71 [1.61; 5.44] |  |  |  |  |  |  |  | 1.81 [0.93; 3.19] | 2.54 [1.00; 5.32] |
| **Trial** |  |  |  |  |  |  |  |  |  |  |  |  |  |  |
| CALGB | 2 | 0 | 0 | 0 | 2 (8.33%) |  | 1 | 1 | 0 | 0 | 0 | 2 | 4 (17.39%) | 6 (12.76%) |
| IALT | 7 | 1 | 2 | 1 | 11 (45.83%) |  | 1 | 2 | 2 | 1 | 1 | 1 | 8 (34.78%) | 19 (40.43%) |
| JBR.10 | 6 | 1 | 4 | 0 | 11 (45.83%) |  | 1 | 2 | 1 | 0 | 0 | 7 | 11 (47.83%) | 22 (46.81%) |
| **Primary NSCLC histology** |  |  |  |  |  |  |  |  |  |  |  |  |  |  |
| Squamous | 9 | 1 | 4 | 1 | 15 (62.50%) |  | 1 | 4 | 2 | 0 | 1 | 6 | 14 (60.87%) | 29 (61.70%) |
| Adenocarcinoma | 5 | 1 | 2 | 0 | 8 (33.33%) |  | 2 | 1 | 1 | 1 | 0 | 2 | 7 (30.43%) | 15 (31.91%) |
| Other | 1 | 0 | 0 | 0 | 1 (4.17%) |  | 0 | 0 | 0 | 0 | 0 | 2 | 2 (8.70%) | 3 (6.38%) |
| **TMB of primary NSCLC** |  |  |  |  |  |  |  |  |  |  |  |  |  |  |
| Low | 4 | 0 | 2 | 1 | 7 (29.17%) |  | 1 | 3 | 2 | 0 | 1 | 1 | 8 (34.78%) | 15 (31.91%) |
| Moderate | 5 | 2 | 2 | 0 | 9 (37.50%) |  | 0 | 1 | 1 | 0 | 0 | 6 | 8 (34.78%) | 17 (36.17%) |
| High | 6 | 0 | 2 | 0 | 8 (33.33%) |  | 2 | 1 | 0 | 1 | 0 | 3 | 7 (30.43%) | 15 (31.91%) |
| **TILs^a^ of primary NSCLC** |  |  |  |  |  |  |  |  |  |  |  |  |  |  |
| Marked | 3 | 0 | 0 | 0 | 3 (12.50%) |  | 0 | 1 | 0 | 0 | 0 | 0 | 1 (4.35%) | 4 (8.51%) |
| Other | 12 | 2 | 6 | 1 | 21 (87.50%) |  | 3 | 4 | 3 | 1 | 1 | 10 | 22 (95.65%) | 43 (91.49%) |

SPC, second primary cancer; NSCLC, non-small cell lung cancer; TMB, Tumor mutational burden; TILs, tumor-infiltrating lymphocytes; Q1, 1st quartile; Q3, 3rd quartile.
Percentages are calculated using the total number of tobacco-related SPCs, non-tobacco-related SPCs, or overall SPCs as the denominator, as appropriate.
^*^ Other includes SPCs arising from localization sites not represented in the predefined LACE-Bio classification (lung, bladder, head and neck, colon and rectum, breast, prostate, esophagus, stomach, pancreas, cervix, small bowel, and Hodgkin disease.
^a^ TIL categories were defined as follows: “marked” = intense lymphocytic infiltration (≥50% stromal lymphocytes); “other” = minimal, mild, or moderate infiltration.

**Supplementary Table 4.** Sensitivity analyses comparing the fully adjusted and parsimonious Fine and Gray competing-risk models for all second primary cancers, tobacco-related and non-tobacco-related SPCs, and death without SPC

|  | **Fully adjusted model** | | | **Parsimonious model** | | |
| --- | --- | --- | --- | --- | --- | --- |
|  | **sHR (95% CI)** | **P value** | **Global P value^*^** | **sHR (95% CI)** | **P value** | **Global P value^*^** |
| **SPC (E = 47)^†^** |  |  |  |  |  |  |
| **TMB** |  |  | 0.88 |  |  | 0.91 |
| Low (E = 15) | 0.83 (0.40-1.75) | 0.63 |  | 0.86 (0.42-1.75) | 0.67 |  |
| Moderate (ref) (E = 17) | 1.00 |  |  | 1.00 |  |  |
| High (E = 15) | 0.91 (0.46-1.83) | 0.80 |  | 0.90 (0.45-1.78) | 0.76 |  |
| TMB (Continuous) | 1.00 (0.99-1.02) | 0.60 | 0.64 | 1.00 (0.99-1.02) | 0.71 | 0.74 |
| Spline (test for non-linearity)^a^ |  | 0.95 |  |  | 0.94 |  |
| **TILs^b^** |  |  | 0.73 |  |  | 0.82 |
| Marked (E = 4) | 0.83 (0.32-2.19) | 0.71 |  | 0.89 (0.34-2.30) | 0.81 |  |
| Other (ref) (E = 43) | 1.00 |  |  | 1.00 |  |  |
| **Tobacco-related SPC (E = 24)^‡^** |  |  |  |  |  |  |
| **TMB** |  |  | 0.91 |  |  | 0.90 |
| Low (E = 9) | 0.80 (0.28-2.33) | 0.69 |  | 0.81 (0.29-2.25) | 0.68 |  |
| Moderate (ref) (E = 7) | 1.00 |  |  | 1.00 |  |  |
| High (E = 8) | 0.91 (0.36-2.30) | 0.84 |  | 0.84 (0.33-2.15) | 0.71 |  |
| TMB (Continuous) | 1.00 (0.98-1.02) | 0.88 | 0.90 | 1.01 (0.98-1.02) | 0.98 | 0.98 |
| Spline (test for non-linearity)^a^ |  | 0.74 |  |  | 0.84 |  |
| **Non-tobacco-related SPC (E = 23)^§^** |  |  |  |  |  |  |
| **TMB** |  |  | 0.98 |  |  | 0.99 |
| Low (E = 8) | 0.91 (0.31-2.70) | 0.87 |  | 0.93 (0.34-2.54) | 0.88 |  |
| Moderate (ref) (E = 8) | 1.00 |  |  | 1.00 |  |  |
| High (E = 7) | 1.01 (0.36-2.79) | 0.99 |  | 0.98 (0.36-2.67) | 0.96 |  |
| TMB (Continuous) | 1.01 (0.99-1.03) | 0.45 | 0.54 | 1.01 (0.99-1.03) | 0.58 | 0.64 |
| Spline (test for non-linearity)^a^ |  | 0.75 |  |  |  |  |
| **Death without SPC (E = 392)** |  |  |  |  |  |  |
| **TMB** |  |  | **0.03** |  |  | **0.03** |
| Low (E = 147) | 1.35 (1.06-1.72) | **0.02** |  | 1.38 (1.09-1.76) | **0.01** |  |
| Moderate (ref) (E = 119) | 1.00 |  |  | 1.00 |  |  |
| High (E = 126) | 1.03 (0.79-1.33) | 0.84 |  | 1.09 (0.84-1.41) | 0.52 |  |
| TMB (Continuous) | 1.00 (0.99-1.00) | 0.25 | 0.23 | 1.00 (0.99-1.00) | 0.26 | 0.26 |
| Spline (test for non-linearity)^a^ |  | 0.17 |  |  | 0.20 |  |
| **TILs^b^** |  |  | 0.08 |  |  | 0.06 |
| Marked (E = 28) | 0.71 (0.46-1.10) | 0.12 |  | 0.70 (0.46-1.06) | 0.09 |  |
| Other (ref) (E = 364) | 1.00 |  |  | 1.00 |  |  |

E, events; SPC, second primary cancer; sHR, subdistribution hazard ratio; ref, reference group; CI, confidence interval; TMB, Tumor mutational burden; TILs, tumor-infiltrating lymphocytes.
Fully adjusted models were stratified by trial, and adjusted for all clinical characteristics (treatment, age, sex, tumor stage, nodal stage, histology, WHO performance status and surgery type).
Parsimonious models were stratified by trial, and adjusted for treatment, tumor stage, nodal stage, and WHO performance status.
^*^ Global P values correspond to likelihood ratio tests.
^a^ Non-linearity was assessed using restricted cubic spline models with three knots.
^b^ TIL categories were defined as follows: “marked” = intense lymphocytic infiltration (≥50% stromal lymphocytes); “other” = minimal, mild, or moderate infiltration. TIL analyses were performed only for the SPC and death without SPC endpoints because of the limited number of tobacco-related SPCs among patients with marked TILs.
^†^ Death without SPC was treated as the competing event.
^‡^ Death without SPC and non-tobacco-related SPCs were treated as competing events.
^§^ Death without SPC and tobacco-related SPCs were treated as competing events.

**Supplementary Table 5.** Baseline characteristics of included and excluded patients

|  | **Total Included**  **(N = 879)** | | **Excluded**  **(N = 29)** | |
| --- | --- | --- | --- | --- |
|  | **N** | ***%^**^*** | **N** | ***%^***^*** |
| **Age** |  |  |  |  |
| ≤ 50 | 161 | *18.32* | 4 | *13.79* |
| 51-60 | 282 | *32.08* | 12 | *41.38* |
| > 60 | 436 | *49.60* | 13 | *44.83* |
| **Sex** |  |  |  |  |
| Male | 640 | *72.81* | 22 | *75.86* |
| Female | 239 | *27.19* | 7 | *24.14* |
| **T-stage*** |  |  |  |  |
| T1 | 109 | *12.40* | 5 | *20.00* |
| T2 | 656 | *74.63* | 20 | *80.00* |
| T3/T4 | 114 | *12.97* | 0 | *0.00* |
| Missing |  |  | 4 |  |
| **N-stage*** |  |  |  |  |
| N0 | 455 | *51.76* | 16 | *64.00* |
| N1 | 298 | *33.90* | 9 | *36.00* |
| N2 | 126 | *14.33* | 0 | *0.00* |
| Missing |  |  | 4 |  |
| **WHO Performance status** |  |  |  |  |
| 0 | 448 | *50.97* | 10 | *38.46* |
| ≥ 1 | 431 | *49.03* | 16 | *61.54* |
| Missing |  |  | 3 |  |
| **Histology** |  |  |  |  |
| Squamous | 410 | *46.64* | 4 | *13.79* |
| Adenocarcinoma | 365 | *41.52* | 10 | *34.48* |
| Other | 104 | *11.83* | 15 | *51.72* |
| **Trials** |  |  |  |  |
| CALGB | 120 | *13.65* | 9 | *31.03* |
| IALT | 507 | *57.68* | 1 | *3.45* |
| JBR.10 | 252 | *28.67* | 19 | *65.52* |
| **Treatment** |  |  |  |  |
| Observation | 438 | *49.83* | 15 | *51.72* |
| Adjuvant chemotherapy | 441 | *50.17* | 14 | *48.28* |
| **Surgery type** |  |  |  |  |
| Pneumonectomy | 279 | *31.74* | 3 | *10.71* |
| Lobectomy/Other | 600 | *68.26* | 25 | *89.29* |
| Missing |  |  | 1 |  |

WHO, World Health Organization.
^*^ 6th Edition TNM staging classification.
^**^ Percentages are calculated using the total number of included patients as the denominator.
^***^ Percentages are calculated after excluding missing values for the corresponding variable.

Supplementary Table 6. Sensitivity analysis of the association between TMB, TILs, and competing endpoints after administrative censoring follow-up at 5 years (fully adjusted Fine and Gray models)

|  | **TMB** | | | **TILs** | | |
| --- | --- | --- | --- | --- | --- | --- |
|  | **sHR^*^ (95% CI)** | **P value** | **Global P value^**^** | **sHR^*^ (95% CI)** | **P value** | **Global P value^**^** |
| **SPC (E = 47)** |  |  |  |  |  |  |
| **TILs^a^** |  |  |  |  |  | 0.92 |
| Marked (E = 4) |  |  |  | 1.05 (0.39-2.82) | 0.91 |  |
| Other (ref) (E = 43) |  |  |  | 1.00 |  |  |
| **TMB** |  |  | 0.66 |  |  |  |
| Low (E = 15) | 1.20 (0.53-2.75) | 0.66 |  |  |  |  |
| Moderate (ref) (E = 17) | 1.00 |  |  |  |  |  |
| High (E = 15) | 0.80 (0.32-1.97) | 0.62 |  |  |  |  |
| TMB (Continuous) | 1.01 (0.99-1.03) | 0.41 | 0.47 |  |  |  |
| Spline (test for non-linearity)^b^ |  | 0.19 |  |  |  |  |
| **Death without SPC (E = 392)** |  |  |  |  |  |  |
| **TILs^a^** |  |  |  |  |  | 0.16 |
| Marked (E = 28) |  |  |  | 0.76 (0.49-1.17) | 0.22 |  |
| Other (ref) (E = 364) |  |  |  | 1.00 |  |  |
| **TMB** |  |  | 0.09 |  |  |  |
| Low (E = 147) | 1.33 (1.03-1.71) | **0.03** |  |  |  |  |
| Moderate (ref) (E = 119) | 1.00 |  |  |  |  |  |
| High (E = 126) | 1.08 (0.83-1.41) | 0.56 |  |  |  |  |
| TMB (Continuous) | 1.00 (0.99-1.00) | 0.38 | 0.37 |  |  |  |
| Spline (test for non-linearity)^b^ |  | 0.33 |  |  |  |  |

TMB, tumor mutational burden; TILs, tumor-infiltrating lymphocytes; E, events; SPC, second primary cancer; sHR, subdistribution hazard ratio; ref, reference group; CI, confidence interval.

^*^ Fine and Gray models were stratified by trial, and adjusted for all clinical characteristics (treatment, age, sex, tumor stage, nodal stage, histology, WHO performance status and surgery type).
^**^ Global P values correspond to likelihood ratio tests.

^a^ TIL categories were defined as follows: “marked” = intense lymphocytic infiltration (≥50% stromal lymphocytes); “other” = minimal, mild, or moderate infiltration.

^b^ Non-linearity was assessed using restricted cubic spline models with three knots.

## 1.2 Supplementary Figures

**Supplementary Figure 1.** Flow diagram of patient selection for the final analytic cohort with complete clinical, TMB and TILs data. TMB, tumor mutational burden; TILs, tumor-infiltrating lymphocytes.

Supplementary Figure 2. Distribution of TMB according to trial, TIL category and histological subtype in the LACE-Bio II study. TMB, tumor mutational burden; TILs, tumor-infiltrating lymphocytes. TIL categories were defined as follows: “marked” = intense lymphocytic infiltration (≥50% stromal lymphocytes); “other” = minimal, mild, or moderate infiltration.

Supplementary Figure 3. Association between tumor mutational burden and the subdistribution hazard of second primary cancer and death without second primary cancer estimated using restricted cubic spline models. Curves are colored according to TMB tertiles. TMB, tumor mutational burden; SPC, second primary cancer.
